# An evaluation of the use of DirectSPR images for proton planning in the RayStation treatment planning software

**DOI:** 10.1002/acm2.13900

**Published:** 2023-01-10

**Authors:** Vikren Sarkar, Adam Paxton, Fanchi Su, Ryan Price, Geoff Nelson, Martin Szegedi, Sara St. James, Bill J. Salter

**Affiliations:** ^1^ University of Utah Salt Lake City Utah USA

**Keywords:** dual energy CT, proton therapy, stopping power ratio, treatment planning

## Abstract

An important source of uncertainty in proton therapy treatment planning is the assignment of stopping‐power ratio (SPR) from CT data. A commercial product is now available that creates an SPR map directly from dual‐energy CT (DECT). This paper investigates the use of this new product in proton treatment planning and compares the results to the current method of assigning SPR based on a single‐energy CT (SECT). Two tissue surrogate phantoms were CT scanned using both techniques. The SPRs derived from single‐energy CT and by DirectSPR™ were compared to measured values. SECT‐based values agreed with measurements within 4% except for low density lung and high density bone, which differed by 13% and 8%, respectively. DirectSPR™ values were within 2% of measured values for all tissues studied. Both methods were also applied to scanned containers of three types of animal tissue, and the expected range of protons of two different energies was calculated in the treatment planning system and compared to the range measured using a multi‐layer ion chamber. The average difference between range measurements and calculations based on SPR maps from dual‐ and single‐energy CT, respectively, was 0.1 mm (0.07%) versus 2.2 mm (1.5%). Finally, a phantom was created using a layer of various tissue surrogate plugs on top of a 2D ion chamber array. Dose measurements on this array were compared to predictions using both single‐ and dual‐energy CTs and SPR maps. While standard gamma pass rates for predictions based on DECT‐derived SPR maps were slightly higher than those based on single‐energy CT, the differences were generally modest for this measurement setup. This study showed that SPR maps created by the commercial product from dual‐energy CT can successfully be used in RayStation to generate proton dose distributions and that these predictions agree well with measurements.

## 1 INTRODUCTION

The use of proton beams for radiation treatment in cancer therapy has become more prevalent during the past decade, with technology evolutions such as pencil beam scanning and single vault solutions helping to drive the increased utilization and access. While much work has been done to increase the accuracy of proton dose calculation algorithms,^1^, ^2^, ^3^, ^4^ the dominant source of inaccuracy remains as uncertainty of the precise range of the dose‐delivering protons. Due to a non‐unique relationship between material composition and the HU assigned to a specific material in a CT dataset, there is ambiguity^5^ in calculating the precise stopping power (SP) of each voxel. Because SP directly determines how far protons will travel in the patient and, thus, where dose will be deposited, uncertainties in SP calculation can lead to potentially critical uncertainties in dose deposition. The traditional approach to dealing with such uncertainties has been to add distal and proximal margins to the target, leading to increased amounts of normal tissue being treated, and an effective undermining of much of the potential advantages of proton beam therapy. Given that one of the major benefits of proton therapy over photon therapy is the lack of exit dose on the distal edge of the target, such methods of range uncertainty mitigation can represent a major challenge in cases where an organ at risk resides near the distal target edge.^6^

With the advent of dual‐energy CT (DECT) scanners, it has now become possible to use the differences in images acquired using two different spectral distributions of x‐rays to obtain more information on the composition of materials within each voxel and, in turn, calculate the stopping‐power ratio (SPR) for that voxel more accurately. Recently a commercial solution (DirectSPR™, Siemens Healthineers, Erlangen, Germany) has become available where the SPR data is calculated from DECT into a purportedly more accurate image set that can then be used for dose planning.

Starting with version 9B, the RayStation (RaySearch Laboratories AB, Stockholm, Sweden) treatment planning system (TPS) has had the ability to use these SPR data sets for proton planning. One of the potential advantages of this possible improvement in SPR representation is that it may allow for a reduction in margins needed for range uncertainty and, thus, more effective exploitation of a key benefit of proton therapy by allowing for tightened distal margins^7^ proximal to down‐range OAR's.

While multiple previous studies^6^, ^8^, ^9^, ^10^, ^11^ have investigated the feasibility of using SPR data generated from DECT for treatment planning, the purpose of this study is to specifically investigate the use of the newly available commercial SPR data within RayStation, and compare the results to the traditional approach of using a single‐energy CT (SECT) for proton planning.

## 2 METHODS

All images in this investigation were acquired on a Siemens Somatom Definition Edge CT scanner (Siemens Healthineers, Erlangen, Germany). For each experimental setup, a single‐energy CT (SECT) scan (120 kVp, 1 mm slice thickness, 50 cm FOV, Br38 reconstruction kernel, iBHC Bone enabled) was obtained. Immediately following this SECT acquisition, a DECT was acquired using energies of 80 kVp and 140 kVp (1 mm slice thickness, 50 cm FOV, Qr40 reconstruction kernel and iBHC Bone enabled). These images were then sent to syngo.via (version VB50) workstation for post‐processing to generate the SPR data. Both the SECT and SPR datasets were then imported into RayStation (version 10A) to create comparison plans.

Conversion from CT value to SPR within the treatment planning system is handled differently for SECT and DECT‐based SPR datasets. For SECT, our clinical conversion curve of CT numbers in HU to mass density, based on a CT scan of a tissue‐equivalent phantom (Advanced Electron Density Phantom, Sun Nuclear Corporation, Melbourne, FL, USA), was used to calculate the mass density of each voxel. Based on the mass density, RayStation then assigns the elemental composition and mean ionization energy to each voxel using a finite number of human tissue materials.^12^ For the DECT‐generated SPR data set, RayStation uses a separate CT conversion curve to convert the raw values stored in the dataset intro SPR values. This curve is only usable when the dataset is appropriately labeled as an SPR dataset in the header information. RayStation will assign the elemental composition and mean ionization number based on the SPR of each voxel, and the corresponding mass density is determined so that the SPR in the dose calculation exactly matches that of the imported SPR value for the proton energy specified in the SPR data set. The stopping power in the RayStation dose computation is computed in real time using the material properties determined from the SECT/SPR data sets using the expression of Bethe and Bloch.^13^ This means that the energy dependence in stopping power is correctly accounted for also for the SPR data set. While the time difference between these processes for handling SECT vs DECT data was not specifically investigated, any such difference was not deemed to have a significant impact on the clinical workflow.

For this investigation, three distinct experiments were performed as described here. For experiments requiring the delivery of proton beams, a Mevion proton therapy system (s250i, Mevion Medical Systems, Littleton, MA, USA) was used.

### 2.1 Investigation of SPR assignment

As a first experiment, SECT and SPR data sets of two phantoms were obtained. The phantoms used were the Advanced Electron Density Phantom (Sun Nuclear Corporation, Melbourne, FL, USA) and Model 062 Electron Density Reference Phantom (CIRS, Norfolk, VA, USA). For each phantom, both datasets were imported into the TPS and rigidly registered to each other. Each tissue‐equivalent insert was contoured on the SECT dataset and copied onto the SPR images. From the SECT dataset, the average CT number in HU within each tissue insert was obtained. The clinical CT‐number‐to‐mass‐density curve was used to convert this to an average mass density, which was then converted to an average SPR using data provided by RaySearch Laboratories. This workflow was meant to reproduce what happens within the TPS whenever SECT is used for proton therapy dose calculations. These values are then compared to the average SPR within the corresponding regions in the SPR data set as well as the SPR for each tissue surrogate plug. The latter was determined experimentally by sending a single beam of protons of two different energies (164.8 MeV and 227 MeV) through the long axis of each plug and measuring the distal 90% range of the exiting proton beam using a Zebra (IBA dosimetry GmbH, Schwarzenbruck, Germany) multi‐layer ion chamber (MLIC).

### 2.2 Comparison of measured and TPS‐calculated beam range

For the second experiment, four 10 × 10 × 15 cm^3^ boxes made from ¼ inch Perspex were filled with water as well as representative tissue samples including ground bovine fat trimmings, ground bovine heart and ground bovine sirloin. SECT and SPR image sets of each box were obtained. Each box was positioned in front of a Zebra MLIC and aligned to machine isocenter using markers placed on each box prior to CT acquisition. A 5 × 5 cm^2^ square field of 227 MeV protons (0.25 cm uniform spot separation) was directed at each box and the position of the distal 90% range (R90d) was obtained. This was repeated using 164.8 MeV protons.

**FIGURE 1:**
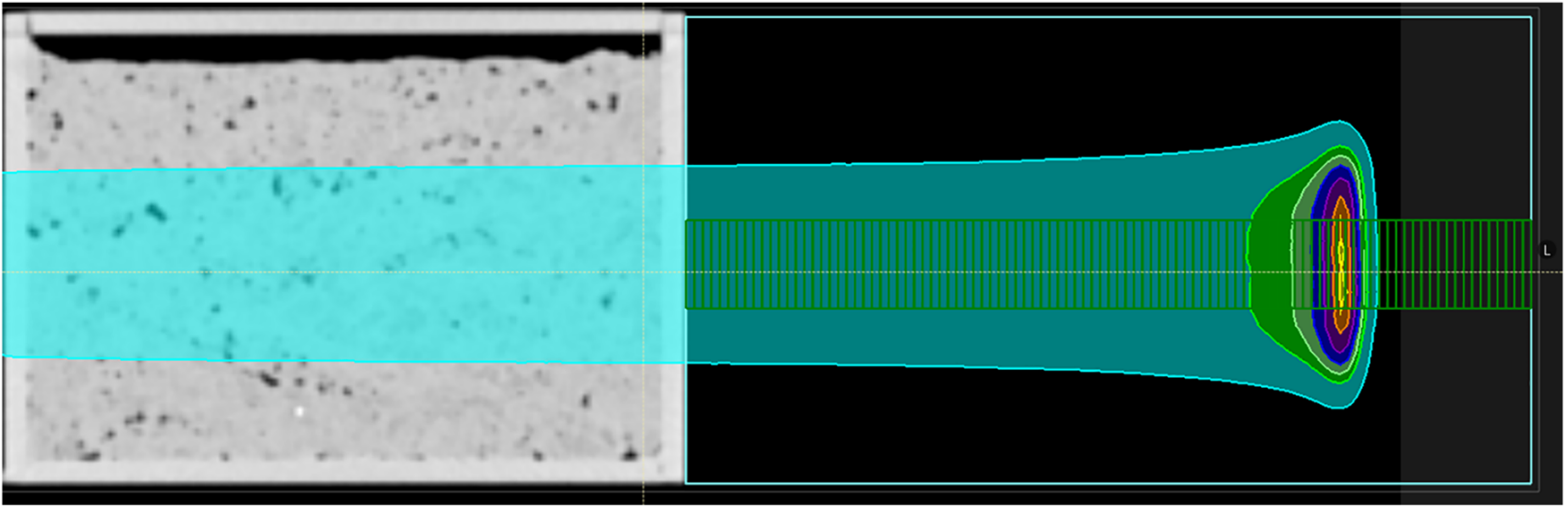
Example phantom setup showing the ion chambers in green that were inserted via a script to sample the dose deposition along the beam direction for a 5 × 5 uniform field of 227.2 MeV protons

SECT and SPR images of all three tissue‐filled boxes were imported into the TPS and treatment plans were created to mimic the proton fields delivered to each of these phantoms. A cuboid region of interest was added on the exit side of the beam and defined as water‐equivalent in the TPS. A script developed in our department was used to add a stack of cylindrical regions of interest (ROI) oriented perpendicularly to the central axis of the beam. Each ROI was made to represent the active volume of the chambers within the MLIC such that a depth dose profile created using the average dose within the stacked ROIs was equivalent to that measured by the Zebra. Figure 1 shows an example of a phantom in RayStation with the stack of ion chambers used to sample the dose.

A second script was used to create this profile in the TPS, which was then processed through a Matlab (version R2021a, Mathworks Inc, Natick, MA, USA) script^14^ to add a Bortfeld fit to the raw depth dose profile. This was done to mirror the workflow within the OmniPro‐Incline software (IBA Dosimetry Gmb, Schwarzenbruck, Germany) when it processes raw data from the Zebra MLIC. This was repeated for all three tissue‐filled boxes, using both the SECT and SPR images for both proton beam energies used. For each case, the TPS‐predicted R90d was then compared to the directly measured value.

### 2.3 Comparison of doses predicted using SECT and DirectSPR data sets to 2D‐array measurements

**FIGURE 2:**
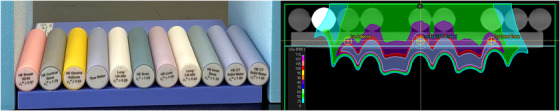
Phantom construction (left) and example dose distribution from the treatment planning system (right)

A set of randomly‐chosen tissue‐equivalent inserts from the Sun Nuclear Advanced Electron Density Phantom was positioned on top of a 30 × 30 × 2 cm^3^ water‐equivalent slab (Solid Water HE, Sun Nuclear Corporation, Melbourne, FL, USA). This phantom is shown in the left panel of Figure 2. SECT and SPR images of this phantom were obtained and used in the TPS to calculate dose distributions (2 mm dose grid resolution and 0.5% uncertainty using the Monte Carlo v5.0 algorithm) using the treatment fields for a randomly selected set of three previously‐treated patients as well as a single low energy (79.8 MeV) 20 × 20 square field of spots uniformly spaced by 2.5 mm and using 0.5 MU per spot. This single low energy was chosen to generate a homogeneous dose distribution at the measurement plane of the 2D array used to measure the distribution. The right panel of Figure 2 shows an example of the dose distribution obtained in the TPS for the square field.

Each of the selected fields were delivered to a 2D ion chamber array (Octavius 1500XDR, PTW Freiburg GmbH, Freiburg, Germany) and compared to both the SECT and SPR predicted doses. In addition to our clinical proton gamma criterion of (3%, 3 mm), the pass rates were also recorded using (1%, 1 mm) and (2%, 2 mm) criteria.

## 3 RESULTS

### 3.1 SPR assignment

Table 1 shows the results of the investigation on SPR assignment for the various tissue‐equivalent plugs within the Sun Nuclear Advanced Electron Density phantom from the SECT and commercial SPR data compared to the measured SPR. Table 2 shows the analogous results for the plugs in the CIRS phantom. For SECT‐based SPR allocation, the SPRs generally agree with measurements within 5% except for low density lung (up to 13% difference) and high density bone (8% difference). For all materials investigated, the SPR obtained by DirectSPR™ agreed with measurements within 2%.

**TABLE 1:** SPR comparison based on SECT and commercial SPR data sets of the Sun Nuclear Electron Density Phantom. The values are compared to the average measured values for each tissue surrogate plug

Material	Vendor specified physical density (g cm^3^)	MLIC Measured rSPR	SECT‐based Assigned rSPR	rSPR from DirectSPR scan	SECT difference from measurement (%)	DirectSPR difference from measurement (%)
Breast	0.98	0.9827	0.9999	0.9887	1.7	0.6
Cortical bone	1.93	1.7012	1.8321	1.7271	7.7	1.5
Adipose	0.96	0.9636	0.9939	0.9767	3.1	1.4
Solid water	1.02	1.0015	1.0105	1.0008	0.9	−0.1
Inner bone	1.21	1.1406	1.1708	1.1491	2.6	0.7
Lung 450	0.45	0.5064	0.4836	0.5026	−4.5	−0.7
Brain	1.05	1.0158	1.0349	1.0230	1.9	0.7
Lung 300	0.29	0.3479	0.3029	0.3424	−12.9	−1.6
Liver	1.08	1.0645	1.0660	1.0510	0.1	−1.3
CaCO3 30%	1.33	1.2570	1.2686	1.2633	0.9	0.5
CaCO3 50%	1.56	1.4285	1.4476	1.4261	1.3	−0.2

**TABLE 2:** SPR comparison based on SECT and commercial SPR image sets of the CIRS Electron Density Phantom. The values are compared to the average measured values for each tissue surrogate plug. The SPR for the Dense Bone plug could not be measured due to the size of the surrogate material relative to the size of the proton beam

Material	Vendor specified physical density (g cm^3^)	MLIC Measured rSPR	SECT‐based Assigned rSPR	rSPR from DirectSPR scan	SECT difference from measurement (%)	DirectSPR difference from measurement (%)
Lung inhale	0.20	0.2181	0.1945	0.2193	−10.8	0.5
Adipose	0.96	0.9843	0.9791	0.9833	−0.5	−0.1
Muscle	1.06	1.0404	1.0557	1.0436	1.5	0.3
Trabecular Bone	1.16	1.1211	1.1369	1.1200	1.4	−0.1
Breast	0.99	1.0048	0.9943	1.0051	−1.0	0.0
Liver	1.07	1.0452	1.0481	1.0490	0.3	0.4
Lung exhale	0.50	0.5349	0.5341	0.5379	−0.1	0.6
Solid water	1.01	1.0542	1.0388	1.0538	−1.5	0.0
Dense bone	1.53	NA	1.4438	1.3912	NA	NA

### 3.2 Comparison of TPS‐predicted range against MLIC‐measured range

Table 3 shows the comparison between the MLIC‐measured range and TPS predictions using the SECT and commercial SPR image sets. For the water comparison, the range differences were similar between SECT and commercial SPR datasets. For the measurements taken using animal tissues, the average difference between measurements and commercial SPR‐based predictions was 0.1 mm while it was 2.2 mm for the SECT‐based predictions. The SECT‐based range predictions were also observed to be consistently lower than the measured range.

**TABLE 3:** Comparison of range measured using an MLIC to TPS range predictions based on both SECT and DirectSPR images

Material	Beam energy (MeV)	MLIC measured range (mm)	Predicted range from SECT scan (mm)	Predicted range from DirectSPR scan (mm)	SECT range difference (mm)	DirectSPR Range difference (mm)
Water	164.8	20.3	20.1	20.5	−0.2	0.2
	227.2	159.3	158.9	159.3	−0.4	0.0
Fat	164.8	30.2	28.4	29.9	−1.8	−0.3
	227.2	169.1	166.7	168.7	−2.4	−0.4
Heart	164.8	20.2	18.3	20.4	−1.9	0.2
	227.2	159.1	156.6	159.2	−2.5	0.1
Muscle	164.8	18.1	16.6	18.2	−1.5	0.1
	227.2	157.4	154.5	156.9	−2.9	−0.5

### 3.3 Comparison of 2D ion‐chamber array measurements to TPS predictions using SECT and DirectSPR images

Table 4 summarizes the various gamma pass rates for the square 20 × 20 field and patient fields. In general, the average gamma pass rates agreed very closely between measurements and either of the two methods of generating dosimetric plans. Only the uniform field (Plan A) showed a slightly lower pass rate for SECT‐based predictions.

**TABLE 4:** Gamma pass rates comparing measurements to SECT‐based and commercial SPR‐based TPS predictions

				SECT‐based plan Gamma Passrate			SPR‐based plan Gamma Passrate		
Plan	Beam	Center Plug	Depth (mm)	(3%,3 mm)	(2%,2 mm)	(1%,1 mm)	(3%,3 mm)	(2%,2 mm)	(1%,1 mm)
A	1	Brain	0	98.4	97.2	83.9	98.4	96.6	75.4
			10	98.4	96.8	74.0	98.4	96.2	78.1
			20	97.8	96.3	81.7	98.4	96.4	82.4
			30	98.4	97.2	85.6	98.4	96.6	86.1
		Bone	0	98.2	95.9	67.7	98.4	95.8	67.0
			10	98.4	95.3	71.1	98.4	95.3	71.3
			20	96.9	91.5	73.1	97.2	91.1	70.8
			30	98.4	96.8	78.8	98.4	95.3	86.5
		Adipose	0	97.3	90.0	62.1	97.3	90.4	72.2
			10	98.4	93.5	74.6	98.4	95.4	72.5
			20	98.1	96.7	73.9	98.1	96.1	71.6
			30	98.4	94.7	71.3	98.4	96.6	89.3
B	1	Brain	20	91.1	73.8	49.3	90.1	74.7	34.0
			40	78.1	63.9	28.9	80.5	60.8	25.8
	2		20	98.4	93.7	79.3	98.4	93.7	74.7
			40	93.6	88.3	46.6	93.6	83.5	38.4
C	1	Brain	20	98.4	97.2	85.6	98.4	96.6	84.5
			50	98.4	97.2	81.1	98.4	96.6	83.6
	2		20	95.7	92.4	78.5	95.8	91.5	79.8
			50	98.4	97.2	94.9	98.4	96.6	92.6
D	1	Brain	10	98.4	95.4	83.6	98.4	95.5	80.1
			50	98.4	97.2	91.4	98.4	96.6	91.1
	2		10	97.8	96.6	96.6	98.4	94.9	79.4
			50	98.4	97.2	97.2	98.4	96.6	94.9
	1	Adipose	10	98.4	94.5	65.7	97.7	94.6	78.8
			40	81.3	52.5	38.9	78.9	57.6	41.2
	2		10	96.1	83.1	54.8	96.7	92.6	58.2
			40	98.4	97.2	95.2	98.4	96.6	92.9
	1	Bone	40	93.8	83.0	60.4	96.1	81.7	57.0
	2		40	87.5	79.8	60.0	90.8	84.6	63.4
Average				95.9	90.7	72.9	96.1	90.9	72.5
Standard deviation				5.1	10.7	17.1	5.0	10.1	17.9

## 4 DISCUSSION

One of the fundamental pieces of data required when calculating a proton dose distribution is the assignment of SPR within each voxel. Previous studies^8^, ^15^, ^16^ have shown that there are systematic differences between SPR assigned from SECT data and SPR data calculated from DECT using different models. The present study confirms this behavior, with the largest differences occurring in tissue surrogates with densities widely different from water, specifically low density lung and high density bone. It should, however, be noted that the differences seen in SECT‐based assignments of SPR using tissue surrogates may not be fully representative of errors seen in real human tissue. Schneider et al.^17^ have previously shown that the type of tissue surrogate used, specifically the chemical composition of these surrogates, strongly affects the HU and SPR assigned to that material, which is why they proposed the stoichiometric method for calibration. The fact that DirectSPR™ values were observed here to yield smaller differences from measurements correlate well with the results of Yang et al.^18^ who showed that DECT‐based SPR determination was much less sensitive to tissue composition than the SECT‐based method.

Our results showed that TPS‐predicted ranges based on SECT and commercial SPR datasets differed by approximately 2 mm for a 15 cm thick target object, or about 1.3% of the depth. In their study on Head and Neck and Pelvic patients by Wohlfahrt et al.^15^ they found a range difference of 1 mm for Head and Neck cases and 4 mm for pelvic treatments. With an assumption that HN treatment depths average 10 cm (range difference = 1%) and pelvic treatment depths average 30 cm (range difference = 1.3%), the observed range difference of 1.3% (2 mm for 15 cm depth) between commercial SPR and SECT predictions agrees well with the Wohlfahrt study.

The results of dose validation using a 2D ion chamber array confirm the accuracy of TPS‐derived relative dose distributions calculated on both SECT and commercial SPR datasets. For all dose distributions validated here, the measurement depth was specifically chosen to be near the distal edge of the dose distributions, where the largest differences have been reported between SECT‐based and DECT‐based predictions. For all of our measurements, the pass rates were comparable even for the very tight (1%, 1 mm) passing criterion. These results differ from those from Mossahebi et al.^6^ where they observed a large change in gamma pass rates between SECT and SPR‐based predictions, even for a very simple plan. We believe that this difference may be due to the fact that their predictions were done in a different treatment planning system and their SPR datasets were from a different source. Another possible difference between our results and Mossahebi could lie in the conversion from CT number to mass density that was used. At least for the SECT‐based predictions, the SPR assigned depends strongly on this curve. Finally, they used a different detector and measured in the middle of the spread‐out Bragg Peak while we took multiple measurements, including at the distal falloff.

While we aimed to make this study as comprehensive as possible, limitations remain that should be discussed. While DirectSPR is enabled for multiple reconstruction kernels, we only investigated one of them (Qr40). We have also not investigated the effect of changing pitch/mAs/slice thickness on the final quality data. While mAs and pitch should not change the image quality as long as the mAs is high enough to give good image quality, the slice thickness could lead to differences. Due to the possibility of motion artifacts being introduced in the DirectSPR data due to the dual energy CTs being acquired at slightly different positions, a deformable image registration step is used between the two datasets as part of the creation of the DirectSPR dataset. A slice thickness greater than 2 mm could lead to inaccuracies in the image registration, leading to differences in the final SPR data assignment for different voxels. This effect was not specifically evaluated in this study. Finally, the tissue samples investigated did not include extremes of density (lung and bone).

## 5 CONCLUSION

The results of this study confirm that DirectSPR™ data sets can be successfully employed within the RayStation TPS to calculate accurate proton dose distributions. A comparison of measured SPR to those based on SECT and DECT of tissue surrogate phantoms confirmed, in the commercial environment studied here, that large differences were observed for SECT‐based values, especially for those surrogates with very low and very high physical densities. These results are consistent with results previously published for other, non‐commercial, environments. Comparison of proton range measurements using animal tissue samples to predictions based on SECT and DECT also supported previous studies, in that DECT‐based measurements were consistently closer to measured range values. DirectSPR™‐based dose distributions agreed more closely with measurements, but 2D array measurements showed that dose distributions calculated using either SECT or DirectSPR™ data sets are comparable when using commonly‐employed comparison metrics, even when measurements were taken at the distal edge of the distributions. Given that the distance metric of the gamma criterion used is larger than the expected range difference between SECT and DECT, this is not a surprising finding.

## AUTHOR CONTRIBUTIONS

Project Conception: V.S., A.P., F.S., R.P.; Data Collection: V.S., A.P., F.S., R.P., S.S., G.N.; Data Analysis: V.S., A.P., F.S., R.P., S.S.; Manuscript Writing: V.S., S.S.; Manuscript Review: All; Other Contributions: M.S., G.N.

## CONFLICT OF INTEREST

None.

## Data Availability

Research data are not shared.
